# Nanopatterning of Perovskite Thin Films for Enhanced and Directional Light Emission

**DOI:** 10.1021/acsami.2c09643

**Published:** 2022-08-09

**Authors:** Loreta A. Muscarella, Andrea Cordaro, Georg Krause, Debapriya Pal, Gianluca Grimaldi, Leo Sahaya Daphne Antony, David Langhorst, Adrian Callies, Benedikt Bläsi, Oliver Höhn, A. Femius Koenderink, Albert Polman, Bruno Ehrler

**Affiliations:** † Center for Nanophotonics, AMOLF, Science Park 104, 1098 XG Amsterdam, The Netherlands; ‡ Department of Chemistry, Utrecht University, Princetonlaan 8, 3584 CB Utrecht, The Netherlands; § Institute of Physics, University of Amsterdam, Science Park 904, 1098 XH Amsterdam, The Netherlands; ∥ Cavendish Laboratory, Cambridge, JJ Thomson Avenue, Cambridge CB3 0HE, United Kingdom; ⊥ Fraunhofer Institute for Solar Energy Systems ISE, Heidenhofstraße 2, 79110 Freiburg, Germany

**Keywords:** halide perovskite, nanopattern, nanoimprint, directional emission, light outcoupling, light emitting diodes, waveguiding modes

## Abstract

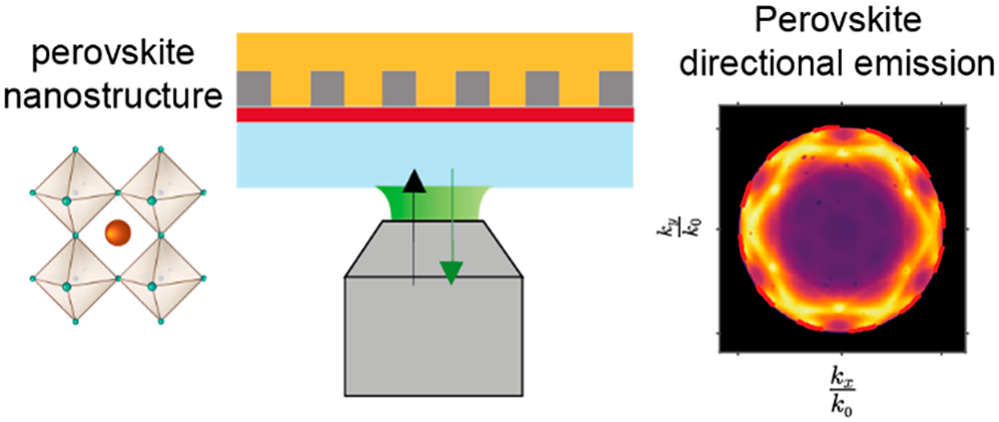

Lead-halide perovskites offer excellent properties for lighting and display applications. Nanopatterning perovskite films could enable perovskite-based devices with designer properties, increasing their performance and adding novel functionalities. We demonstrate the potential of nanopatterning for achieving light emission of a perovskite film into a specific angular range by introducing periodic sol–gel structures between the injection and emissive layer by using substrate conformal imprint lithography (SCIL). Structural and optical characterization reveals that the emission is funnelled into a well-defined angular range by optical resonances, while the emission wavelength and the structural properties of the perovskite film are preserved. The results demonstrate a flexible and scalable approach to the patterning of perovskite layers, paving the way toward perovskite LEDs with designer angular emission patterns.

## Introduction

Lead-halide perovskites have recently gathered significant attention due to their intriguing optoelectronic properties and easy fabrication that make them suitable for a plethora of applications, e.g., in photovoltaics,^1^ lighting,^2^ thermoelectric materials,^3^ and photodetectors.^4^ For solid-state lighting applications, perovskite-based light emitting diodes (PeLEDs) offer easy bandgap tunability from the visible to the infrared achieved via low-cost and low-temperature solution-based compositional engineering,^5^ high color purity of the emission,^6^ and high photoluminescence quantum yield (PLQY).^7−9^ One of the most important metrics to quantify LED performance is the external quantum efficiency (EQE).^10^ The EQE depends on the product of the carrier injection efficiency, the PLQY, and the light outcoupling efficiency. Carrier injection efficiency and PLQY can be very high in optimized PeLEDs, for example, by mitigating nonradiative charge carrier recombination (e*.*g. via antisolvent engineering^11^ or incorporating chemical additives^12−15^). The EQE is limited by the outcoupling efficiency of the light generated in the perovskite layer. The light outcoupling efficiency is defined as the ratio of photons flux coupled out of the emissive layer to the photon flux generated within the emissive layer. Due to the large mismatch in the refractive index between the perovskite^16−18^ and the hole/electron injection layers,^19^ most of the light generated in the film is trapped in the substrate or in the active layer by excitation of waveguide modes, leading to a light outcoupling efficiency of only ≈20%.^17,20^ Reduction of the internal reflection of the generated photons can be achieved by random patterning (i.e., enhancing the roughness of the substrate) and nanopatterning (i.e., introducing periodic structures) in the charge transport layers or in the emissive layer, leading to increased light outcoupling and therefore directly boosting the device efficiency.^21−23^ Opposite to random patterning, nanopatterning enables directional light emission with designer properties, allowing for light emission into a specific angular range or/and with a desired polarization. For instance, such properties could be applied in an adaptive automotive for smart headlights, which actively switch the radiation pattern in response to road situation and hazards, in building-integrated photovoltaics to enhance the absorption at specific angles or in angle-sensitive photodetectors that can perform optical-domain spatial filtering, extending the edge enhancement capabilities of these devices. Polarization in lighting could be relevant to reduce glare, e.g., in car-headlight situations. Several mask-free (e.g., inkjet and e-jet printing, laser-assisted patterning, laser ablation) and mask-assisted (e.g., nanoimprint, template-confined patterning by photolithography) methods have been employed to obtain periodically structured substrates.^24−26^ However, only a few strategies have been successfully applied to perovskite layers.^27−34^ Despite the many potential routes for obtaining periodically patterned perovskites, the fabrication of perovskite LEDs with custom angular emission properties remains challenging, as this would require both flexibility in the design of the periodic structures, precision in the fabrication, and scalability of the approach. In principle, imprinting the perovskite directly using a polydimethylsiloxane (PDMS) mold would fulfill these requirements. However, due to the hardness of the perovskite film compared to that of the liquid polymeric materials typically imprinted, vapor-assisted decomposition and regrowth of the perovskite are simultaneously required during the direct imprint.^35^ This decomposition-regrowth process has been demonstrated to affect the crystallization, which in turn affects the optoelectronic properties of the emissive layer. In addition, due to the low volatility of the precursors in the inorganic perovskites (e.g., CsPbBr_3_), this approach can be used only for organic-based perovskites. Planar hot pressing has also been used to pattern as-deposited perovskite layers that are recrystallized following the pattern shape by thermal imprint.^36,37^ However, patterning strategies that require the application of external pressure on the perovskite layer might change the material properties due to the mechanically soft and dynamically disordered perovskite lattice. In addition, inhomogeneity in the pressure exertion might result in varying local properties.

Substrate conformal imprint lithography (SCIL)^38^ is a promising nanopatterning strategy, which combines low-cost fabrication, reusability of the stamp, and the potential of large-area patterning,^38^ while still achieving sub-10 nm feature resolution.^39^ This approach does not limit the choice of the hole injection layers (i.e., both inorganic and organic transport layers can be used) and the type of perovskites adopted in device configurations (i.e., inorganic and organic). In addition, as only the hole/electron injection layer is imprinted directly, the possibility of pressure-induced inhomogeneity during the perovskite growth and local property variations are avoided. Furthermore, the lifetime of the stamp is significantly extended in comparison with nanopatterning strategies on harder materials where the stamp lifetime is limited to a few fabrication iterations.

In this work, we introduce periodic structures between the hole injection layer and the emissive layer (i.e., the perovskite) by patterning a silica sol–gel layer via SCIL. The subsequently spin-coated perovskite film infills and overgrows the pattern. The photoluminescence of the patterned film shows an increase of two to three times in the emission intensity and a dramatic change in the angular distribution of the emission, while the other properties of the material remain mostly unchanged. The versatility, high control, and modularity of the patterning process allows for the design of custom structures, enabling a wide range of light management strategies, from maximizing light outcoupling to controlling the angular emission of the active layer, while avoiding damage to the active material.

## Experimental Section

### Materials

Indium tin oxide (ITO) coated glass substrates and borosilicate substrates were purchased from KINTEC. Anhydrous *N*,*N*-dimethylformamide (DMF, 99% purity), anhydrous dimethyl sulfoxide (DMSO, 99.9% purity), lead bromide (PbBr_2_, trace metals basis), poly(9-vinylcarbazole) (PVK, *M*_n_ 25000–50000), lithium fluoride (LiF, ≥ 99.99% trace metals basis), and chlorobenzene (CB, > 99.8% purity) were purchased from Sigma-Aldrich. Methylamine hydrobromide (MABr, > 98% purity) was purchased from TCI. T1100 sol–gel was purchased from SCIL Nanoimprint Solution. 2,2′,2″-(1,3,5-Benzinetriyl)-tris(1-phenyl-1-*H*-benzimidazole) (TPBi, > 99.5% purity) was purchased from Ossila. Aluminum pellets (Al, 99.99% purity) were purchased from Kurt Lesker.

### Preparation of MAPbBr_3_ and PVK Solutions

DMF and DMSO were mixed in a 4:1 (DMF/DMSO) volume ratio. The solvent mixture is used to prepare stock solutions of PbBr_2_ and MABr by dissolving these precursors at 0.5 M. The PbBr_2_ solution is stirred at 100 °C for 1 h to fully dissolve the powder. MAPbBr_3_ solutions were prepared by mixing the MABr with PbBr_2_ stock solutions at 1:1 molar stoichiometric ratios (i*.*e., 1:1 v/v). The MAPbBr_3_ solution was stirred at 50 °C until the MABr powder was fully dissolved in the PbBr_2_ solution. For the PVK (hole injection layer) solution, 15 mg/mL PVK powder was dissolved in anhydrous chlorobenzene in a nitrogen-filled glovebox. The solution was stirred overnight at room temperature.

### PVK Deposition

The half-stack (HS) and the full-stack (FS) include a layer of PVK between the borosilicate glass (or ITO) substrate and the sol–gel. For those stacks, the PVK solution is deposited by spin coating in a cleanroom at 1000 rpm for 60 s (1000 rpm/s acceleration), resulting in a film of 25 nm thickness. The films are annealed at 120 °C on a hot plate for 20 min. A short treatment using an Oxford Instrument’s PlasmaPro 80 and a process employing O_2_ flow (25 sccm) at 10^–6^ mTorr pressure at ignition for 30 s is applied to increase the wettability of the surface before the deposition of the next layer. Each oxygen plasma treatment removes 5 nm from the initial PVK layer.

### Sol–Gel Deposition and Imprint

Borosilicate glass (or ITO) substrates are cleaned with soap and sonicated for 20 min in water, 20 min in acetone, and 20 min in isopropanol. T1100 sol–gel is deposited by spin coating on the borosilicare or ITO at 2000 rpm for 8 s (acceleration 2000 rpm/s). To imprint the sol–gel, we used flexible large-area rubber stamps made out of a quaternary siloxane-modified polydimethyl-siloxane (PDMS) material with a high Young’s modulus. The PDMS layer is laminated to a thin glass carrier (200 μm), providing high in-plane stiffness while maintaining out-of-plane flexibility. More detailed information on SCIL, including attainable resolution, stamps layers, and process, is reported other works.^38,39^ After spin coating, the silica sol–gel layer is approximately 75 nm thick. During imprint, the liquid sol–gel is pushed away by the PDMS features. After curing (7 min), the sol–gel used in this work becomes mostly SiO_2_ and the residual silica layer is approximately 20 nm while the imprinted features are ∼100 nm, which equals the etch dept of the Si master from which the stamp is molded. The sol–gel residual layer at the bottom of the imprinted holes is cleared via reactive ion etching using an Oxford Instrument’s PlasmaPro 80 and a process employing CHF_3_ (25 sccm) and Ar (25 sccm) (etch time 2 min 30 s, etch rate 0.28 nm/s). This etching step removes the silica in between the imprinted features,

### MAPbBr_3_ Deposition

A short treatment using Oxford Instrument’s PlasmaPro 80 and a process employing O_2_ flow (25 sccm) at 10^–6^ mTorr pressure at ignition for 30 s is applied to increase the wettability of the surface before the deposition of the perovskite layer. The perovskite is spin coated at 4500 rpm for 30 s in a nitrogen filled glovebox. After 10 s, 200 μL of CB is dropped onto the sample to induce the perovskite crystallization. After the deposition, the perovskite layer is annealed at 100 °C for 15 min.

### Electron Transport Layer and Electrode Deposition

In the FS configuration, TPBi is deposited by thermal evaporation at a pressure of 7.7 × 10^–7^ mbar at a rate of 0.5 Å/s for a total thickness of 40 nm. LiF (1.2 nm) is thermally evaporated at a rate of 0.1 Å/s. Finally, 70 nm of Al, used as the top electrode, is evaporated at a rate of 1 Å/s. The samples for thermal evaporation are loaded into the evaporator in a nitrogen filled glovebox.

### Characterizations

The XRD pattern of thin films was measured using a X-ray diffractometer, Bruker D2 Phaser, with Cu Kα 1.5406 Å as the X-ray source, 0.02° (2θ) as the step size, and 0.10 s as the exposure time. The measurements were performed from 2° to 65° (2θ). The slit used was 0.6 mm wide and the knife height was 0.5 mm. A FEI Verios 460 was used to perform electron microscopy. An electron beam acceleration of 7 kV in high vacuum and 100 pA as the current were the settings used for the measurement. The sample was measured at 0° and 90° relative to the beam, to probe the surface and the cross-section, respectively. Time-correlated single-photon counting (TCPSC) measurements of the investigated samples were performed with a home-built setup equipped with PicoQuant PDL 828 “Sepia II” and a PicoQuant HydraHarp 400 multichannel picosecond event timer and TCSPC module. A 485 nm pulsed laser (PicoQuant LDH-D-C-640) with a repetition rate of 10 MHz was used to excite the sample. A Thorlabs FEL-420 long-pass filter was used to remove the excitation laser. A home-built inverted microscope setup was used for Fourier microscopy, where the sample is mounted with the glass side face down and both pump and fluorescence signals are guided through the same objective (Nikon, CFI L Plan EPI CRA, 100×, NA 0.85, glass corrected) and same side, i.e., from the glass side. The sample is pumped using a 532 nm pulsed laser (Teem Photonics, type STG-03E-1S0), which has a pulse width of 400 ps and a maximum energy per pulse of 3.5 μJ. The measurements were performed at a laser repetition rate of 1 kHz at a pump pulse energy of 4.5 nJ. An epi lens is placed in the pump path, resulting in a parallel beam of diameter around 65 μm at the sample plane. Fluorescent light is separated from the pump light using a dichroic mirror and further filtered of unwanted pump reflection light by a long-pass filter (cut off wavelength ∼540 nm) before detection. Fluorescence is detected by either a thermoelectrically cooled (Andor Clara) Si CCD camera or a Shamrock 303i spectrometer (grating 1:300 lines/mm) with an (Andor Ivac) Si CCD detector. The detector is selected by flipping a mirror. To focus the light onto the CCD or spectrometer entrance slit (slit width opening is 50 μm), we used a tube lens of 200 mm focal length. The Andor IVAC camera contains a CCD chip with 200 × 1650 pixels. A commercial inverted microscope (Nikon) was used for the photoluminescence measurements, with the sample glass side down, and both pump and fluorescence occur through the same objective (Nikon, CFI L Plan EPI CRA, 100×, NA 0.85, glass corrected) and same side, i*.*e., from the glass side. The sample is epi-illuminated (around 45 μm^2^ spot size) using a 450 nm pulsed laser (LDH-P-C-450B, Pico Quant), which has a pulse width of <70 ps at a repetition rate of 40 MHz. The fluorescence from the sample is detected by the camera (PCO. edge 4.2, PCO AG) in a spectrometer (PI Acton SP2300).

## Results and Discussions

**Figure 1:**
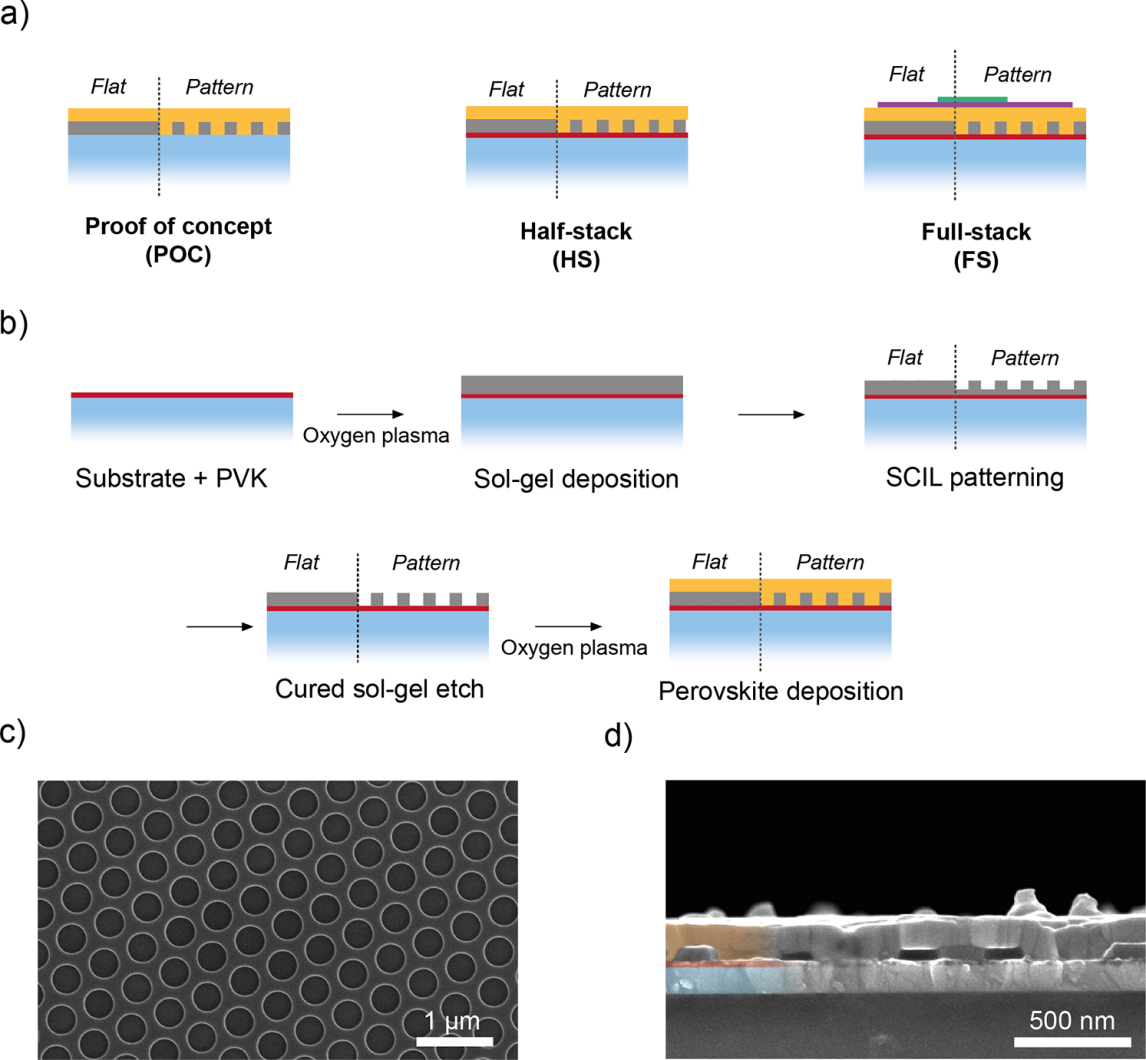
(a) Three configurations used in this work: proof of concept (POC), half-stack (HS), and full-stack (FS). Substrate, PVK, sol–gel, perovskite, TPBi, LiF, and Al areas are in blue, red, gray, orange, purple, and green, respectively. (b) Schematic of the SCIL technology used for the fabrication of patterned MAPbBr_3_ thin films. (c) SEM image showing the successful patterning of the sol–gel on top of the PVK (after the sol–gel etch, the PVK will be exposed in the holes) with the desired design and (d) the intrusion of the perovskite (orange) into the hexagonal sol–gel pattern (gray) deposited on ITO (blue) and PVK (red).

The samples discussed in this work are named as proof of concept (POC), half-stack (HS) and full-stack (FS) configuration (Figure 1a). The POC configuration consists of borosilicate/sol–gel/MAPbBr_3_. The HS configuration consists of borosilicate/PVK/sol–gel/MAPbBr_3_, where the PVK is used as hole-injection layer. The FS configuration consists of borosilicate/PVK/sol–gel/MAPbBr_3_/TPBi/LiF/Al, where the TPBi and LiF/Al are used as electron-injection layers and top electrode, respectively. PVK, TPBi, LiF, and Al were chosen because they are the most common hole- and electron-injection layers and top electrodes used in green-emitting PeLEDs. To reduce sample-to-sample variations induced by the spin coating and etching process, half of the sol–gel on our borosilicate substrate is not imprinted (i*.*e., flat) and the other half is imprinted (i.e., patterned). Thus, the same perovskite spin coating and etching process is experienced by both the flat and patterned area on the same substrate. This fabrication strategy is applied to all the configurations.

Figure 1b illustrates the SCIL fabrication strategy for both the flat and patterned area of the substrate, including the PVK layer used in the HS configuration. PVK is deposited on the substrate as described in the Experimental Section. After the short oxygen plasma treatments (before the sol–gel and perovskite deposition), the final thickness of the PVK is ∼15 nm. Afterward, a 70 nm thick sol–gel layer is spin-coated on the PVK layer, and the stamp is applied to the wet sol–gel on half of the substrate. After a curing time of about 7 min, the stamp is carefully removed. The use of silica sol–gel is very convenient as opposed to other imprintable resists. After curing, the sol–gel used in this work becomes SiO_2_^39^ (∼90 wt% silicon oxide and remaining organic components), which is an excellent hard mask for most of reactive ion etching processes. In addition, the refractive index of cured sol–gel is very close to that of SiO_2_ with negligible losses. Finally, the cured sol–gel is electrically insulating, enabling the charge transfer in the HS configuration only via PVK. The successful imprint of the sol–gel layer on top of the PVK is confirmed by scanning electron microscopy (SEM) images, as shown in Figure 1c. The resulting hexagonal array consisting of a period of 534 nm alternates holes in the cured sol–gel layer that are uniform in size (170 nm in radius and ∼70 nm in depth) across a large area on the centimeter scale (Figure S1a). Sol–gel reactive ion etching and oxygen plasma treatments are then performed to remove the residual sol–gel in the holes and to activate the PVK surface before the perovskite deposition, respectively. The oxygen plasma treatment leads to the removal of ∼5 nm of the PVK layer, whereas the sol–gel reactive ion etching does not affect the thickness of the PVK. The final thickness of the PVK in the patterned stack after the above-mentioned etching steps is ∼15 nm, as in the flat area of the substrate. Thus, the precursor solution of MAPbBr_3_ is spin-coated on the substrate, covering both the flat and the patterned areas. Figure 1d demonstrates the successful infiltration of the perovskite into the patterned sol–gel. In each configuration, the thickness of the perovskite is ∼200 nm on the flat cured sol–gel area, ∼135 nm on top of the cured sol–gel structures, and ∼70 nm within the holes of the pattern, resulting in a total thickness of ∼200 nm as in the flat cured sol–gel. The perovskite infiltrates the pattern, resulting in a smooth and almost planar top interface. We identify some irregular features on top of the perovskite layer that could be dust particles together with clusters of perovskite, possibly formed during the spin-coating process. To avoid charging and drifting effects, SEM images of the imprinted sol–gel before and after the perovskite deposition are collected on ITO substrates. Eventually, to fabricate the FS configuration suitable for green emitting PeLEDs we completed the stack by evaporating the electron injection layers, TPBi and LiF, and Al as the top electrode (Figure S1b). The details of the fabrication process are reported in the Experimental Section.

**Figure 2:**
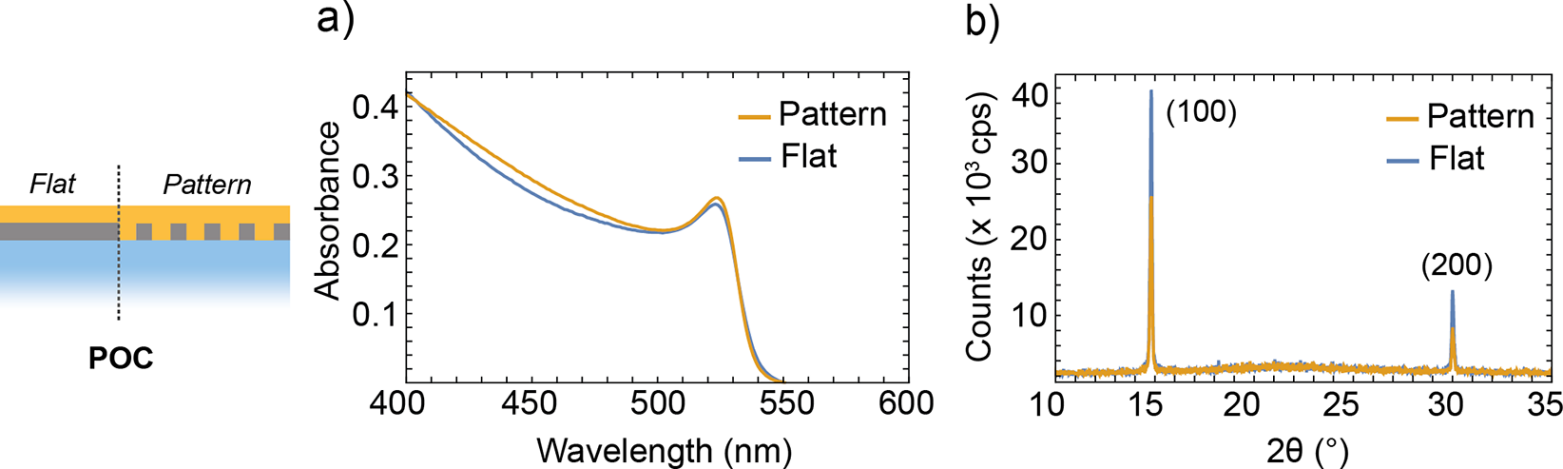
(a) Absorbance of MAPbBr_3_ in the POC configuration on the flat and patterned areas. (b) X-ray diffractogram of MAPbBr_3_ in the POC configuration on the flat and patterned cured sol–gel showing the typical (100) and (200) reflections for the cubic structure of MAPbBr_3_.

The nanopattern may have an influence on the optical and structural properties of the perovskite material induced for instance by the larger surface area or by additional strain during the crystal growth. To assess the impact of the nanostructured substrates on the perovskite growth and on its optical properties, we performed UV–vis absorption spectroscopy and X-ray diffraction (XRD) on the POC configuration, both on the flat and patterned areas. UV–vis absorption measurements reported in Figure 2a, show that the position of the characteristic excitonic peak of MAPbBr_3_ is unchanged in both the flat and patterned areas and it peaks at ∼536 nm (2.31 eV). The perovskite deposited on the flat and the patterned areas also exhibits very similar optical density around the excitonic peak. As we expect the total perovskite volume to be the same for the two geometries, this indicates that the absorbances in the two systems are comparable. X-ray diffractograms shown in Figure 2b show two peaks at 14.8° and 30.0°, which correspond to (100) and (200) lattice planes in MAPbBr_3_, respectively. These features are present both in the flat and patterned areas of the POC configuration and can be assigned to a cubic (*Pm*3*m*) crystal structure of MAPbBr_3_ according to previous reports.^40^ A zoom-in of the two overlapping peaks, normalized to the (110) peak, is reported in Figure S2. We find no change in the peak position or width for the two areas. Whereas the intensity of the peaks is different in the flat and patterned areas, the intensity ratio between the two peaks is the same. Therefore, we argue that the crystal growth of MAPbBr_3_ perovskite on the patterned cured sol–gel is comparable to the one on the flat cured sol–gel, further demonstrating the compatibility of the proposed fabrication scheme with standard perovskite PeLEDs processing. Additionally, the roughness of the perovskite grown on the flat and patterned areas are comparable for each configuration as reported in Figure S3 and Table S1.

**Figure 3:**
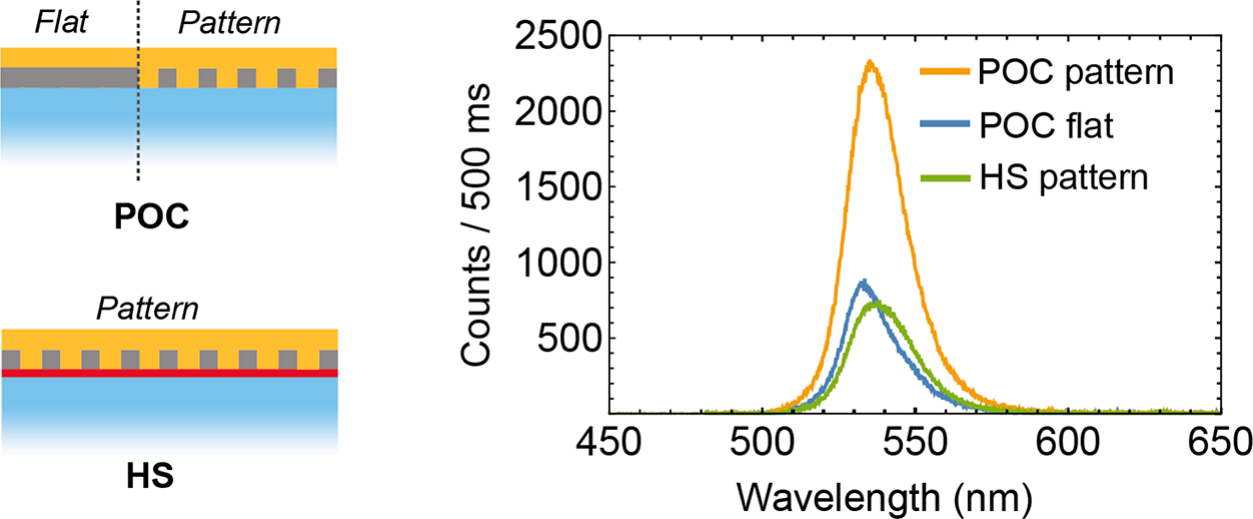
Photoluminescence of the perovskite in the POC configuration on the flat area (blue curve), on the patterned area (orange curve), and on the patterned area in the HS configuration (green curve). The perovskite is excited at a wavelength of 445 nm and 40 MHz excitation frequency.

To evaluate the effect of the nanopattern on the emission properties of MAPbBr_3_, we collect the photoluminescence of the perovskite in the POC configuration. PL measurements reveal a comparable emission wavelength at 532 and 535 nm for the perovskite grown on the flat and patterned areas, respectively. However, we observe a 2.5-fold intensity increase on the patterned sol–gel (Figure 3, orange curve) compared to that on the flat area (Figure 3, blue curve). The presence of the PVK at the perovskite interface is expected to extract charge carriers that are otherwise available for emission reducing the photoluminescence intensity as observed in Figure 3 (green curve). The enhancement of the perovskite emission grown on the patterned area could be attributed to several factors that are discussed in the following: (i) an enhanced radiative emission rate, (ii) trap passivation effects favored by larger contact between the sol–gel and the perovskite film, and (iii) enhanced light-outcoupling efficiency in the out-of-plane direction.

**Figure 4:**
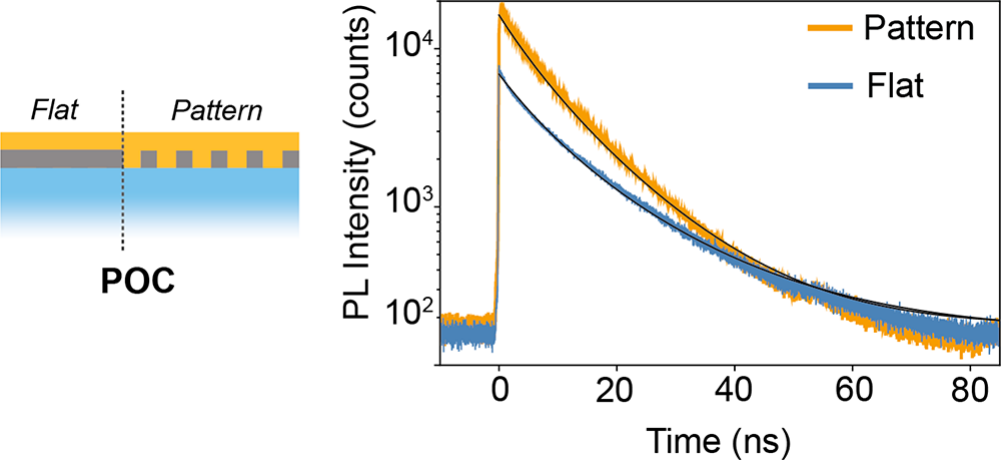
Time-resolved photoluminescence decay of flat and patterned areas of (80 × 80) μm^2^ in the POC configuration on the flat (blue curve) and patterned (orange curve) areas, excited at a wavelength of 485 nm and 10 MHz excitation frequency. The fit is shown in solid black lines.

To study the effect of the pattern on the emission and recombination rates, we performed time-resolved photoluminescence measurements on the POC configuration using time-correlated single photon counting (TCSPC), exciting the flat and patterned area of the sample from the perovskite side at a wavelength of 485 nm with a repetition rate of 10 MHz. The absolute and the normalized photoluminescence decay traces of a flat and a patterned (80 × 80) μm^2^ area in the POC configuration are reported in Figure 4 and Figure S4, respectively. The curves are fitted using the following model^41,42^
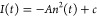
1where *c* is a background signal, *A* is a scaling factor, and *n* is the density of photoexcited carriers, evolving according to the rate equation
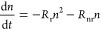
2where *R*_r_ is the radiative decay rate and *R*_nr_ is the nonradiative decay rate. This model does not include Auger recombination, since this contribution is negligible at the excitation density used (<10^19^ photons/cm^3^). We collected photoluminescence decay traces of three distinct maps of the flat and patterned areas. The averaged nonradiative decay rates for the flat and patterned area are (2.01 ± 0.01) and (2.9 ± 0.3)10^7^ s^–1^, respectively. The averaged radiative decay rates for the flat and patterned areas are (6.50 ± 0.04) and (5.4 ± 1.2)10^–8^ cm^3^ s^–1^, respectively. The radiative and the nonradiative decay rates of both the flat and the patterned areas are similar between each other within the error and the spot-to-spot variations (Figure S3). Furthermore, a fast decay component in one of the patterned areas required the inclusion in the model of an additional exponential term (see Note S1). The similar decay rates observed for the flat and patterned areas suggest that the photoluminescence enhancement in the patterned area cannot be attributed to changes in the nonradiative or in the radiative decay rate due to passivation. Such passivation^43^ effects would lead to smaller nonradiative decay rates, which is opposite to what is observed for the perovskite grown on the patterned area. Therefore, these findings suggest a strong contribution from improvements in light outcoupling efficiency.

**Figure 5:**
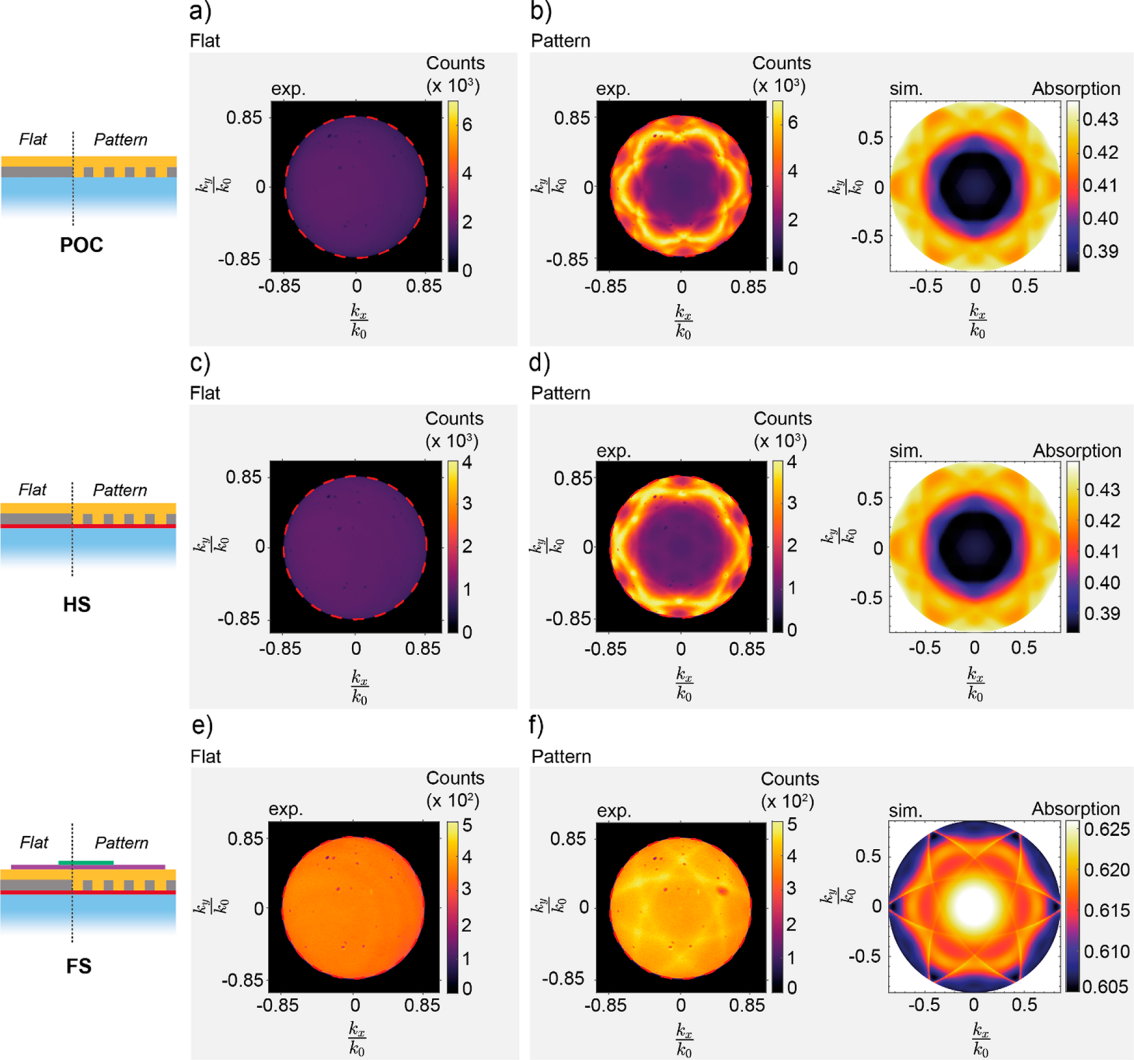
Angle-resolved photoluminescence intensity maps of the perovskite on the (a) flat and (b) experimental and simulated patterned areas in the POC configuration, (c) flat and (d) experimental and simulated patterned areas in the HS configuration, and (e) flat and (f) experimental and simulated patterned areas in the FS configuration. The excitation and the collection of the emission were performed from the glass side. Borosilicate is pictured in blue, PVK in red, sol–gel in gray, perovskite in yellow, TPBi in purple, and LiF/Al in green. Dashed red lines in the Fourier images represent the 0.85 NA objective collection area.

We argue that the improved outcoupling originates mainly from change in the angular distribution of the perovskite PL emission, which reduces the fraction of light coupled into guided modes by total internal reflection. To corroborate this hypothesis, we performed Fourier microscopy to measure the angle-dependent photoluminescence at normal incidence illumination. The samples are epi-illuminated from the glass side with a 532 nm pulsed laser, and PL emission is collected in the same direction as the pump and through the same objective. We collected the PL emission at wavelengths longer than 540 nm, employing a long-pass filter in the detection path to block the pump light. The detailed setup description is provided in the Experimental Section. Fourier imaging maps the objective’s back focal plane onto a CCD camera, hence providing quantitative information on the distribution of emission intensity over all angles within the objective numerical aperture (NA = 0.85). Fourier images are panchromatic images, i.e., not resolved in spectral components, thus the intensity shown is averaged over all the wavelengths (from 540 to 700 nm). Figure 5 shows such angle-resolved photoluminescence intensity maps for the POC, HS, and FS configuration both on the flat (Figure 5a,c,e) and patterned (Figure 5b,d,f panels) sol–gel areas. The outer edge of these Fourier images is set by the numerical aperture (NA) of the air objective used in the measurement. We used a 0.85 NA objective, which allows for a maximum collection angle of ∼58° in air.

In the POC configuration (Figures 5a,b), we observed a uniform angular emission from the perovskite deposited on the flat sol–gel, as expected. We observed a 3-fold enhancement in emission intensity in perovskite on the patterned area compared to the flat area. This enhancement is consistent with the PL measurements shown in Figure 3. Quite remarkably, the patterned counterpart exhibits high-intensity emission at large angles, close to the outer edge of the angular observation range, compared to the normal direction. The presence of sharp features at high angles indicates that light is emitted in specific directions and follows a hexagonal symmetry. These preferential angular bands are dictated by the diffraction grating conditions provided by the pattern periodicity and effective mode index of the emissive layer, as explained in the following.

Waveguiding can occur in a dielectric layer when that layer has a higher refractive index than the adjacent media,^44−50^ with the number of possible modes being dependent on the layer thickness and index. In our case, the perovskite layer acts as a waveguide, hence creating emission into higher in-plane momentum vectors. This is due to its higher refractive index (*n* ≈ 2.1–2.3)^51,52^ compared to the organic injection layers (*n* ≈ 1.6–1.76)^53^ and transparent electrodes (*n* ≈ 1.8–2.2).^54^ Thus, the emitted light will be trapped in the perovskite layer, resulting in a low outcoupling efficiency, as observed for the perovskite grown on the sol–gel flat area. The sol–gel periodic pattern acts as a diffraction grating. Any periodic structure upon plane wave excitation with an in-plane wavevector **k**_||_^in^ scatters the incoming light out into diffraction orders with momentum **k**_||_^out^ by altering the in-plane momentum according to the Bragg equation as **k**_||_^out^ = **k**_||_^in^ + **G**. The term **G** represents the reciprocal lattice vector whose magnitude is given by , where *m* is an integer and *d* denotes the pattern’s periodicity. Thus, the pattern scatters out the waveguide mode, which populates a ring of in-plane wavevectors of radius |**k**_||_^in^|/*k*_0_ = *n*_mode_ (where *n*_mode_ is the waveguide mode index and *k*_0_ the free-space wavenumber) controllably into specific directions to the far field.^44,45^ Therefore, emitted light is outcoupled more efficiently from the perovskite grown on patterned structures and the angular emission pattern is drastically changed. To further corroborate that the pattern is responsible for this drastic change, we mapped out the dispersion diagram of the emission by imaging a slice of the Fourier image centered at *k*_*x*_ = 0 onto the slit of the imaging spectrometer in full imaging mode, as shown in Figure S6 in Note S3. The spectrally resolved Fourier image is a direct map of the dispersion diagram.

By virtue of reciprocity,^44,55^ it is possible to calculate the radiated power into the far-field due to a dipole in the near field by simulating the near field in response to far-field driving. This principle allows us to compare angle-resolved absorption to angle-resolved emission into the far-field. We find good qualitative correspondence between the experimental angle-resolved emission and its simulated counterpart. Figure 5b (panel simulations) shows the angular distribution of the absorbed light incident from the far-field in the POC configuration. As reported in Note S2, this also reflects the simulated angular emission.

Fourier images of the perovskite grown in the HS configuration, which includes the PVK as the hole-injection layer, are shown in Figure 5c,d. The PL intensity of the perovskite on the flat sol–gel area is comparable to the one observed in the POC configuration. This is expected because the insulating flat sol–gel acts as a barrier for charge transfer from the perovskite to the PVK, thus no change in the PL intensity should occur. However, the PL intensity of the perovskite on the patterned area shows a decrease in counts, suggesting charge transfer to the PVK while the strong directional emission is preserved. We note that the light in the simulated structure for the HS configuration (Figure 5d) is emitted predominately at higher angles as compared to the normal direction.

Finally, in the FS configuration, which includes layers typical in PeLEDs (Figure 5e,f), we observe a different angular emission pattern with less evident but still clear features superimposed on a uniform background in the case of the patterned substrate. We observe only a 1.2-fold enhancement in the emission intensity of the perovskite on the patterned area compared to that of the flat one. We attribute this reduced enhancement to the larger roughness of the top contact surface in comparison to the POC and HS configuration as shown in Figure S3 and Table S1 and to the charge transfer in the presence of both hole and electron transport layers In the simulated structure of the FS configuration (Figure 5f), the presence of the top contact modifies the emission from going mostly to steep angles to largely in the normal direction. Nonetheless, sharp features indicating enhanced directional emission are still visible. Finally, we note that material losses and losses related to morphological imperfections (nonuniform pattern, roughness in the layers, etc.) obviously might limit the device performance and reduce the effect of the pattern.

## Conclusion

To conclude, we have successfully fabricated MAPbBr_3_ perovskite thin films on nanopatterned substrates by using SCIL. We have experimentally demonstrated enhanced outcoupling and structured angular emission. This approach introduces periodic sol–gel structures between the injection and emissive layers by patterning a layer of silica sol–gel. We have shown that the perovskite intrudes the cured sol–gel pattern and grows in the desired configuration preserving the bulk structural and optical properties observed for the one grown on the flat cured sol–gel area. Furthermore, we have observed a 3-fold enhancement in the photoluminescence of the active layer in the presence of the sol–gel pattern. Our results reveal that the enhanced photoluminescence observed for the patterned film cannot be ascribed to enhancement in pump absorption or better surface passivation but rather is due to increased light-outcoupling induced by the nanophotonic structure. The latter acts as a diffraction grating redirecting emitted light that is initially trapped in guided modes of the perovskite slab toward narrow angular ranges in free space. This was confirmed by optical characterization of the angular photoluminescence distribution, which could be described qualitatively by simulations of the optical modes in the structures. The use of SCIL as a fabrication method for nanostructured perovskite films opens exciting new opportunities for light management in optoelectronic devices. Indeed, through careful design of optical resonances, it is possible to boost the efficiency also of solar cells and photodetectors. Moreover, the results presented here pave the way for perovskite-based LEDs with designer structured emission and functionalities that go beyond conventional lighting.

## Supporting Information Available

Figures of photographs of patterned perovskite-based light emitting diode, magnified X-ray diffractograms, AFM images of POC, HS, and FS configuration, normalized photoluminescence decay traces, fitting model for photoluminescence decay traces, and dispersion plots as a function of energy and **k**_||_, table of RMS roughness of the perovskite in the POC and HS configuration, and details for simulations of the emission patterns (PDF)
