# Supplementary material for: Nanopatterning of Perovskite Thin Films for Enhanced and Directional Light Emission
Source: ACS Appl Mater Interfaces. 2022 Aug 9;14(33):38067–76. doi: 10.1021/acsami.2c09643 (PMC9412957; doi:10.1021/acsami.2c09643)
Supplement: Supplementary file 1 — am2c09643_si_001.pdf [file am2c09643_si_001.pdf]

Supporting Information for

Nanopatterning of Perovskite Thin Films for Enhanced and Directional Light Emission

Loreta A. Muscarella,[1,2,†] Andrea Cordaro,[3,1,†] Georg Krause,[1] Debapriya Pal,[1] Gianluca Grimaldi,[1,4] Leo Sahaya Daphne Antony,[1] David Langhorst,[1] Adrian Callies,[5] Benedikt Bläsi,[5] Oliver Höhn,[5] A. Femius Koenderink,[1,3] Albert Polman,[1] Bruno Ehrler[1,*]

† The authors have contributed equally

*Corresponding author

b.ehrler@amolf.nl

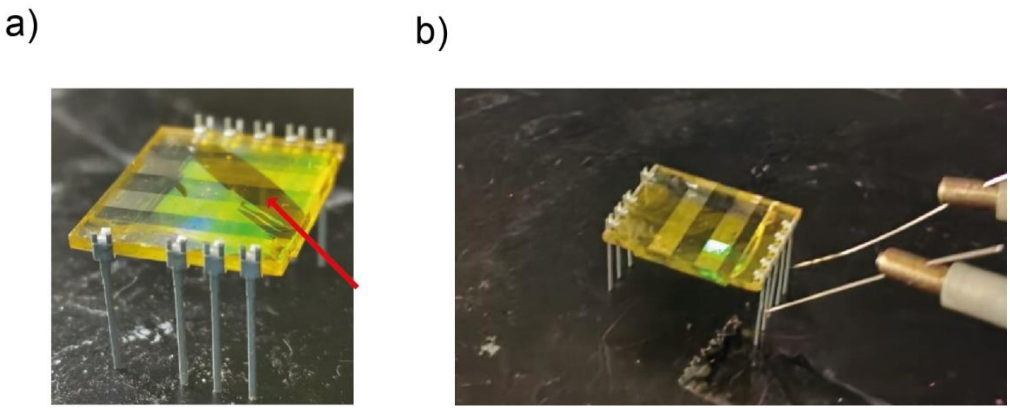

Figure S1. a) A perovskite-based light emitting diode in the FS configuration, ITO/PVK/sol-gel/pattern/perovskite/TPBi/LiF/Al. The patterned area extends to the whole substrate surface area (1.5x2.0 cm). The dark green area indicated by the red arrow represents a pattern-free region. **b)** The same device shows the typical green electroluminescence upon application of a continuous voltage bias.

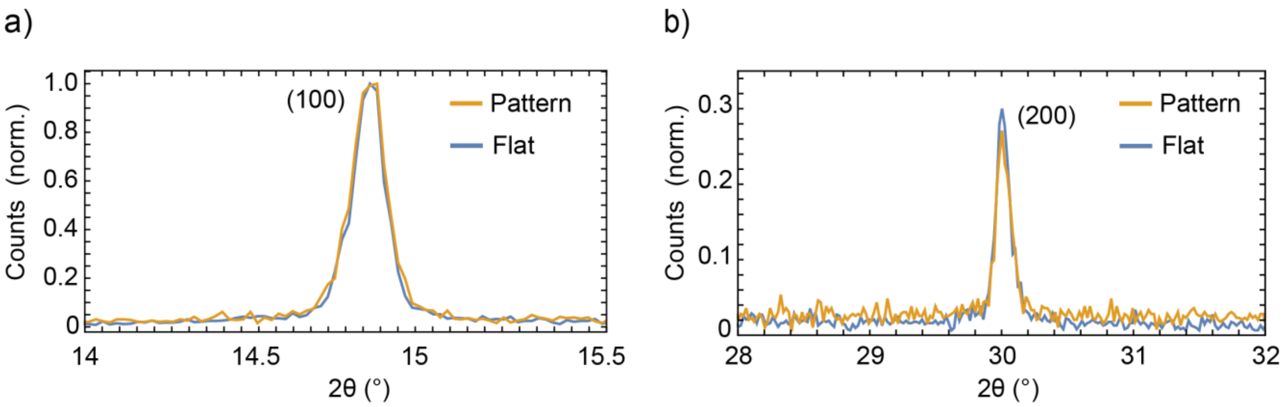

Figure S2. X-Ray diffractogram (λ_{Cu}= 1.5406 Å) of $MAPbBr_3$ grown on flat and patterned areas in the POC configuration with **a)** magnification of the (100) and **b)** (200) reflection showing no change in the peak position and width. Both peaks are normalized to the (100) peak height.

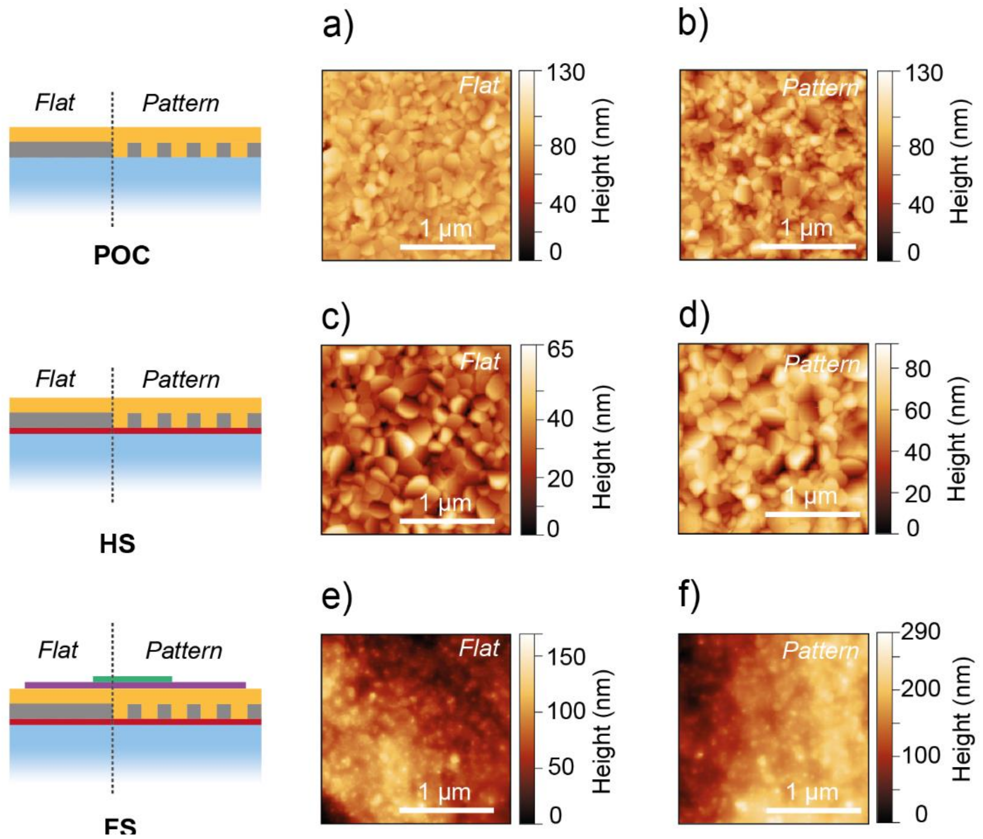

Figure S3. AFM images of the perovskite in the POC configuration on the **a)** flat and **b)** patterned area, in the HS configuration on the **c)** flat and **d)** patterned area. AFM images of the top layer in the FS configuration (*i.e.*, Aluminium) on the **e)** flat and **f)** patterned area.

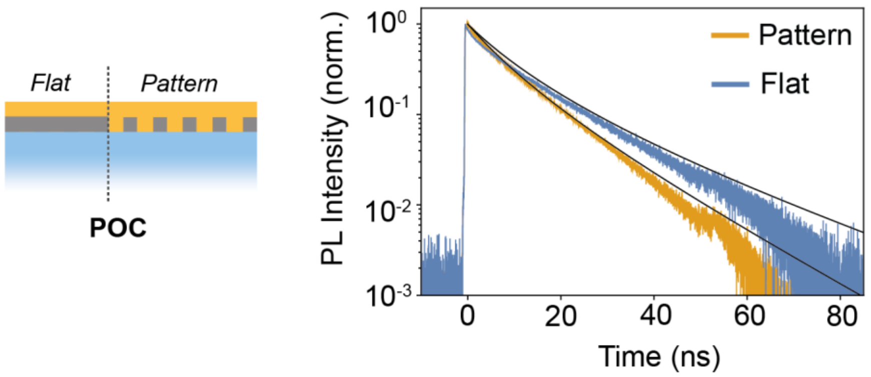

Figure S4. Normalized time-resolved photoluminescence decay in logarithmic scale of a flat and patterned area of (80x80) μm^2 in the POC configuration on the flat (blue curve) and patterned (orange curve) area, excited at a wavelength of 485 nm and 10 MHz excitation frequency. The fit is shown in solid black lines.

Table S1. RMS roughness of the perovskite in the POC and HS configuration, and of the aluminium layer in the FS configuration on the flat and patterned area

**Configuration**	**Flat**	**Patterned**
POC	(10±1) nm	(12±1) nm
HS	(9±1) nm	(13±1) nm
FS	(26±1) nm	(52±1) nm

Supplementary Note 1

For the spots measured on the flat stack and two of the spots measured on the patterned stack deposited on borosilicate glass, the photoluminescence decay curves are fitted using the function described in the main text. For the third spot measured on the patterned stack, an additional empirical exponential term is included into the fitting function to describe the decay rate, R_{extra} , at early times as follows

$$I(t) = A(n_{model}(t))^2 + A_{extra}e^{(-t\,R_{extra})} + c$$

indicating with n_{model} the solution of the rate equation shown in the main text. We speculate that this additional component might originate from an additional decay pathway at this spot, potentially by a large local trap state density. **Figure S5** shows the radiative and non-radiative decay rates of the spot measured in the patterned stack and the associated spot-to-spot variation. The averaged non-radiative decay rates for the flat and patterned stack are $(2.01\pm 0.01)10^{7}$ s^{-1} and $(2.9\pm 0.3)10^{7}$ s^{-1}, respectively. The averaged radiative decay rates for the flat and patterned stack are $(6.50\pm 0.04)10^{-8}$ cm^3 s^{-1} and $(5.4\pm 1.2)10^{-8}$ cm^3 s^{-1}, respectively.

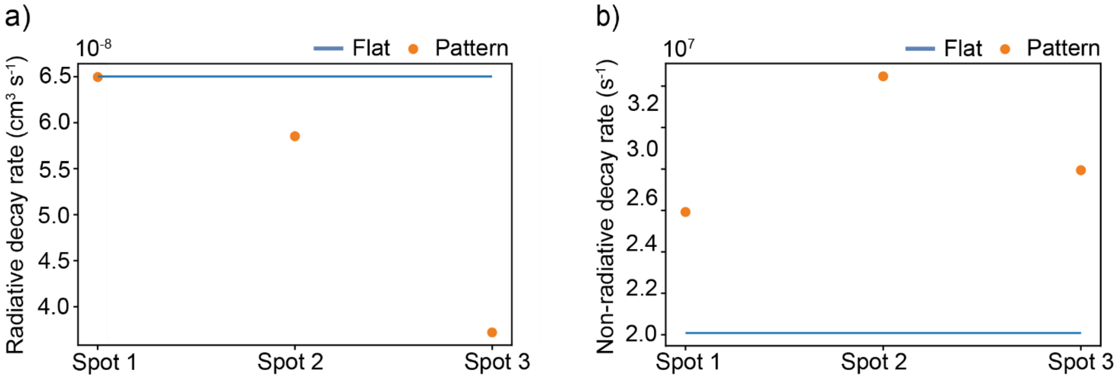

Figure S5. a) Radiative and **b)** non-radiative decay rates of the perovskite in the flat stack and in three areas of the patterned stack.

Supplementary Note 2

We use rigorous coupled wave analysis (RCWA) to numerically simulate the emission patterns of the various structures as they would appear in absorption.[1] The RCWA simulations were done with the program S4, a freely available software by Liu and Fan.[2] We used a truncation to 100 plane waves at a fixed wavelength of 540 nm. In the simulation, we set the refractive index of perovskite,[3,4] sol-gel, PVK,[5] TPBi,[5] LiF/aluminium,[6] and the borosilicate substrate[7] as 2.15 + 0.15j, 1.42, 1.56, 1.8, 0.912 + 6.55j, 1.47, respectively. We take the thickness of the perovskite layer, TPBi/LiF, PVK, Al metal as 135nm, 40nm, 20nm, 70nm, respectively. We assume the dimension of the solgel grating structure as mentioned in the main text. Using these values in the simulation setup, we calculate angle-resolved emission patterns for different patterned layered structures as shown in **Figure 5** in the main text.

Supplementary Note 3

As described in the main text, we measure and plot the dispersion diagram of emission for different structure configurations, as shown in **Figure S6**. For plane waves propagating in the super/substrate, the wave vector $k_{\|}$ equates to $(k_x, k_y) = \frac{2\pi}{\lambda} n \sin(\theta)\ (\cos\phi, \sin(\phi)$ with l the vacuum wavelength, n the refractive index of the super/substrate, θ the angle of propagation relative to the substrate normal, and ϕ the azimuthal angle of propagation. When alluding to the length of the parallel wavevector we mean $k_{\|} = \sqrt{k_x^2 + k_y^2}$. For guided modes the use of the angle q is of course not pertinent - in this case the length of the parallel momentum is given by $\frac{2\pi}{\lambda} n_{mode}$. where instead of the material refractive index the waveguide mode index n_{mode} appears. Thus, the correlation between $k_{\|}$reported in **Figure S6** and k_x and k_y reported in **Figure 5** is $k_{\|} = \sqrt{k_x^2 + k_y^2}$.

The measurements in the left panel are from flat structures, whereas the right panel is for its patterned counterpart for identical layer configurations. We can observe two distinct features in these measurements. First, we observe bright features above ~ 2.2 eV as expected from the PL band emission of the perovskite layer. Second, the prominent emission lines at certain $k_{\|}$ are the diffracted emission from the grating structure in the case of patterned structures, in contrast to the regular emission pattern in flat structures.

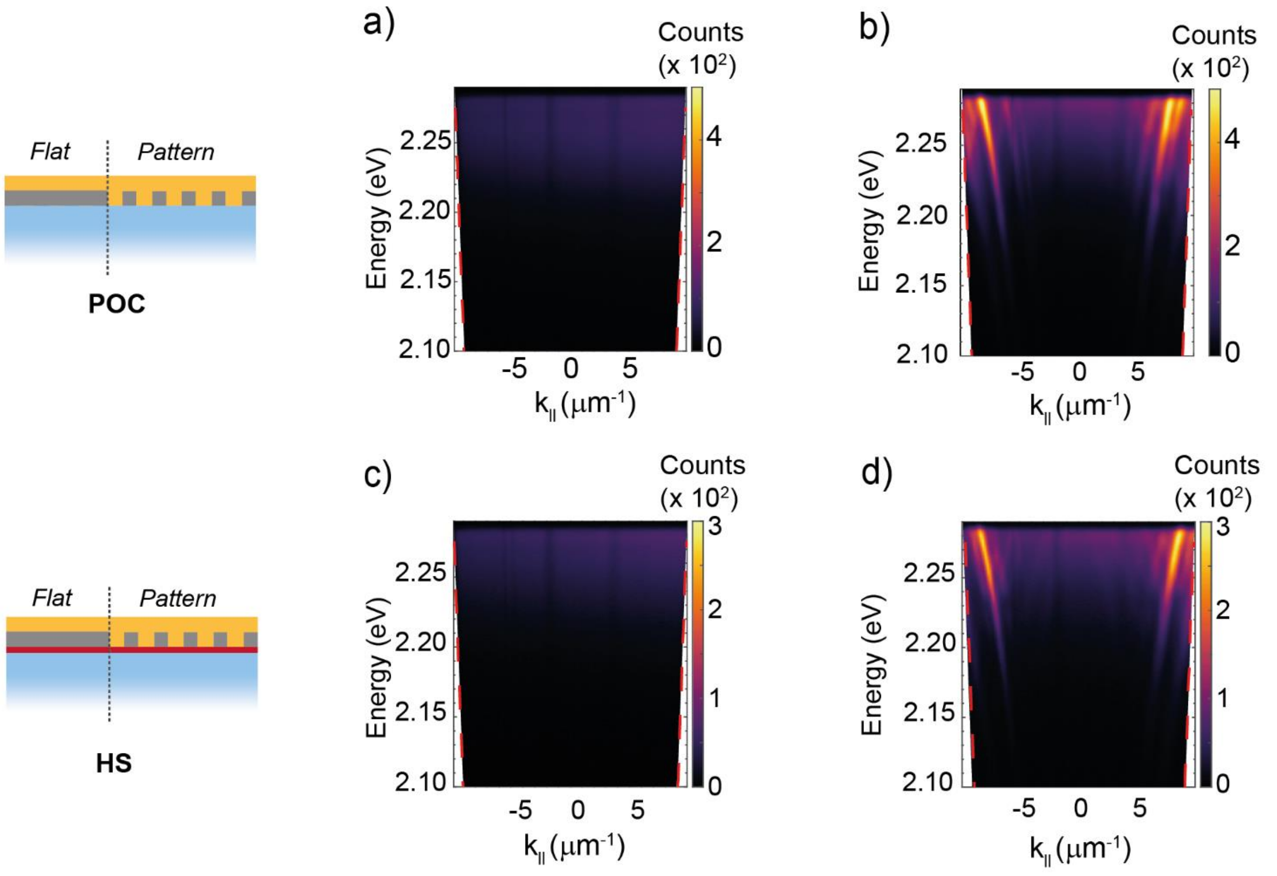

Figure S6. Measured photoluminescence dispersion plots of perovskite as a function of energy and $k_{\|}$ on **a)** flat and **b)** patterned are in the POC configuration, and on a **c)** flat and **d)** pattern area in the HS configuration.

The excitation and the collection are from the glass side. Dashed purple lines represent the 0.85 NA objective collection area.

References

1. Vaskin, A.; Kolkowski, R.; Koenderink, A. F.; Staude, I. Light-Emitting Metasurfaces. *Nanophotonics* **2019**, *8* (7), 1151–1198.
2. Liu, V.; Fan, S. S 4: A Free Electromagnetic Solver for Layered Periodic Structures. *Comput. Phys. Commun.* **2012**, *183* (10), 2233–2244.
3. Rigneault, H.; Lemarchand, F.; Sentenac, A.; Giovannini, H. Extraction of Light from Sources Located inside Waveguide Grating Structures. *Opt. Lett.* **1999**, *24* (3), 148.
4. Langguth, L.; Schokker, A. H.; Guo, K.; Koenderink, A. F. Plasmonic Phase-Gradient Metasurface for Spontaneous Emission Control. *Phys. Rev. B - Condens. Matter Mater. Phys.* **2015**, *92* (20), 2054015.
5. Lozano, G.; Rodriguez, S. R. K.; Verschuuren, M. A.; Rivas, J. G. Metallic Nanostructures for Efficient LED Lighting. *Light: Science and Applications*. Nature Publishing Group, **2016**, e16080–e16080.
6. Rakić, A. D. Algorithm for the Determination of Intrinsic Optical Constants of Metal Films: Application to Aluminum. *Appl. Opt. 7* **1995**, *34* (22), 4755–4767.
7. Technical details of BOROFLOAT® https://www.schott.com/en-nz/products/borofloat-p1000314/technical-details?tab=24f18f4c9fd845f4bf8f6968328233b4.
